# Stability and Oligomerization of Mutated SMN Protein Determine Clinical Severity of Spinal Muscular Atrophy

**DOI:** 10.3390/genes13020205

**Published:** 2022-01-24

**Authors:** Emma Tabe Eko Niba, Hisahide Nishio, Yogik Onky Silvana Wijaya, Mawaddah Ar Rochmah, Toru Takarada, Atsuko Takeuchi, Tomokazu Kimizu, Kentaro Okamoto, Toshio Saito, Hiroyuki Awano, Yasuhiro Takeshima, Masakazu Shinohara

**Affiliations:** 1 Department of Community Medicine and Social Healthcare Science, Kobe University Graduate School of Medicine, 7-5-1 Kusunoki-cho, Chuo-ku, Kobe 650-0017, Hyogo, Japan; niba@med.kobe-u.ac.jp (E.T.E.N.); yogik.onky@gmail.com (Y.O.S.W.); mashino@med.kobe-u.ac.jp (M.S.); 2 Department of Occupational Therapy, Faculty of Rehabilitation, Kobe Gakuin University, 518 Arise, Ikawadani-cho, Nishi-ku, Kobe 651-2180, Hyogo, Japan; 3 Department of Neurology, Faculty of Medicine, Public Health and Nursing, Universitas Gadjah Mada, Jalan Farmako, Sekip Utara, Yogyakarta 55281, Indonesia; mawaddah.arr@gmail.com; 4 Laboratory of Functional Molecular Chemistry, Kobe Pharmaceutical University, 4-19-1 Motoyamakitamachi, Higashinada-ku, Kobe 658-8558, Hyogo, Japan; takarada@kobepharma-u.ac.jp; 5 Instrumental Analysis Center, Kobe Pharmaceutical University, 4-19-1 Motoyamakitamachi, Higashinada-ku, Kobe 658-8558, Hyogo, Japan; takeuchi@kobepharma-u.ac.jp; 6 Department of Pediatric Neurology, Osaka Women’s and Children’s Hospital, 840 Murodo-cho, Izumi 594-1101, Osaka, Japan; kimizu@wch.opho.jp; 7 Department of Pediatrics, Ehime Prefectural Imabari Hospital, 4-5-5 Ishii-cho, Imabari 794-0006, Ehime, Japan; kentaro206@gmail.com; 8 Department of Neurology, National Hospital Organization Osaka Toneyama Medical Center, 5-1-1 Toneyama, Toyonaka 560-8552, Osaka, Japan; saito.toshio.cq@mail.hosp.go.jp; 9 Department of Pediatrics, Kobe University Graduate School of Medicine, 7-5-2 Kusunoki-cho, Chuo-ku, Kobe 650-0017, Hyogo, Japan; awahiro@med.kobe-u.ac.jp; 10 Department of Pediatrics, Hyogo College of Medicine, 1-1 Mukogawa-cho, Nishinomiya 663-8501, Hyogo, Japan; ytake@hyo-med.ac.jp

**Keywords:** SMA, mutated SMN1 protein, stability, oligomerization

## Abstract

Spinal muscular atrophy (SMA) is a common autosomal recessive neuromuscular disease characterized by defects of lower motor neurons. Approximately 95% of SMA patients are homozygous for *survival motor neuron 1* (*SMN1*) gene deletion, while ~5% carry an intragenic *SMN1* mutation. Here, we investigated the stability and oligomerization ability of mutated SMN1 proteins. Plasmids containing wild- and mutant-type *SMN1* cDNA were constructed and transfected into HeLa cells. Reverse transcription-polymerase chain reaction (RT-PCR) demonstrated similar abundances of transcripts from the plasmids containing *SMN* cDNA, but Western blotting showed different expression levels of mutated SMN1 proteins, reflecting the degree of their instability. A mutated SMN1 protein with T274YfsX32 exhibited a much lower expression level than other mutated SMN1 proteins with E134K, Y276H, or Y277C. In immunoprecipitation analysis, the mutated SMN1 protein with T274YfsX32 did not bind to endogenous SMN1 protein in HeLa cells, suggesting that this mutation completely blocks the oligomerization with full-length SMN2 protein in the patient. The patient with T274YfsX32 showed a much more severe phenotype than the other patients with different mutations. In conclusion, the stability and oligomerization ability of mutated SMN1 protein may determine the protein stability and may be associated with the clinical severity of SMA caused by intragenic *SMN1* mutation.

## 1. Introduction

Spinal muscular atrophy (SMA) is an autosomal recessive neurodegenerative disease characterized by defects of lower motor neurons in the spinal cord, resulting in weakness and wasting of voluntary muscles [1]. SMA is arguably the most common genetic disorder, with an incidence of approximately 1 in 6000 to 20,000 live births and a carrier frequency of 1/40–1/70 in the general population [2,3,4,5].

SMA is clinically divided into five groups: type 0 (the most severe form with onset in the prenatal period; severe respiratory problems after birth), type 1 (a severe form with onset before 6 months of age; unable to sit unsupported), type 2 (an intermediate form with onset before 18 months of age; able to sit unaided, but unable to stand or walk), type 3 (a mild form with onset after 18 months of age; able to stand and walk unaided), and type 4 (the mildest form with age of onset from adolescence to adulthood) [6].

The *survival motor neuron* (*SMN*) genes located on chromosome 5q13 [7], *SMN1* and *SMN2*, were identified as being related to SMA in 1995 [8]. *SMN1* and *SMN2* are nearly identical genes with only five nucleotide differences between them: one in intron 6, one in exon 7, two in intron 7, and one in exon 8 [8]. These differences do not lead to changes of the encoded amino acid sequence. 

However, the gene expression patterns of *SMN1* and *SMN2* are different. *SMN1* produces only full-length (FL) *SMN1* transcript and FL-SMN1 protein. In contrast, *SMN2* produces only small amounts of FL-*SMN2* transcript and FL-SMN2 protein [9,10]. Overall, 90% of the transcripts produced by *SMN2* lack exon 7 (exon 7 skipping; ∆7 *SMN2* transcript) due to the conversion of C to T at position 840 in exon 7. The protein product from the ∆7 *SMN2* transcript is a ∆7 SMN2 protein lacking the C-terminal domain, which is shortened, unstable, and rapidly degraded [2,6]. The rapid degradation of ∆7 SMN2 protein may be due to the lack of oligomerization ability [11] and the creation of a degradation signal (degron) [12].

Homozygous deletion of *SMN1* is identified in 95–97% of SMA patients, while intragenic mutation is found in the remaining 3–5% [4,13,14,15]. Some of the mutations that give rise to SMA are small insertions, deletions, and occasionally missense mutations in the *SMN1* gene [14,15]. These subtle mutations are usually found in one *SMN1* allele in the patients, with deletion or conversion in the other *SMN1* allele. To the best of our knowledge, only two reports of subtle mutation occurring in a homozygous state have been published [16,17].

It has been reported that increased *SMN2* copy number might be related to improved survival outcomes and maintenance of motor function in SMA patients, suggesting that FL-SMN2 protein compensates for the loss of *SMN1* to some degree [6]. In fact, a genotype–phenotype correlation of greater *SMN2* copy number being associated with lower SMA severity has been shown in SMA patients with the complete absence of *SMN1*. Thus, *SMN1* is now considered to be the disease-causing gene, while *SMN2* is the disease-modifying gene [2,6,15]. 

However, in patients with an intragenic *SMN1* mutation, the genotype–phenotype correlation is not clear [18,19]. This is because dysfunction of the mutated SMN1 protein may contribute more to the clinical severity than the *SMN2* copy number [15,19,20,21]. We have already suggested two possible factors determining the clinical severity of SMA: one is complex formation with partner proteins bound to the mutated SMN1 protein [20] and the other is stability of the mutated SMN1 protein [22]. SMN is known to self-oligomerize, and certain mutations in the C-terminal region disrupt oligomerization. Disruption of oligomerization hampers SMN function in small nuclear ribonucleoprotein (snRNP) assembly [23,24,25]. Additionally, SMN binds to Gemin 2–7 and Sm proteins to form a multimeric protein complex [23]. Oligomerization and multimeric complex-formation abilities modulate the stability of SMN protein [26]. 

In this study, to clarify the factors determining the clinical phenotype of patients with intragenic *SMN1* mutation, we attempted to estimate the stability and oligomerization ability of the mutated SMN1 proteins. Here, we finally suggested that the interaction of mutated SMN1 protein with FL-SMN2 protein may determine the clinical severity of such SMA patients. 

## 2. Materials and Methods

### 2.1. Patients and Their Intragenic SMN1 Mutations

Five intragenic mutations were identified in six patients. All of the patients had been reported elsewhere [19,20,22,27,28,29,30], and clinical information on each patient is summarized in Table 1. Mutations of Patients 1, 2, 4, 5, and 6 were analyzed in our laboratory. Patient 3 with c.400 G > A mutation (p.Glu134Lys substitution) was not one of our own patients [30], but was included in this study as a typical case of mutated Tudor domain; the disease severity and the results of molecular genetic analysis were reported previously [27,30].

Prior to this study, informed consent was obtained from all patients analyzed in our laboratory. All procedures were reviewed and approved by the Ethics Committee of Kobe University Graduate School of Medicine (reference number 1089, approved on 5 October 2018), and were conducted in accordance with the World Medical Association Declaration of Helsinki.

### 2.2. Outline of the Experiments

To analyze the stability of mutated SMN1 proteins, (1) we constructed plasmids containing wild-type and mutant-type *SMN1* cDNA, and (2) transfected them into HeLa cells. (3) Mutated *SMN* transcripts and SMN1 proteins expressed in HeLa cells were determined by RT-PCR and Western blotting.

To elucidate the oligomerization ability of mutated SMN1 proteins with an intragenic mutation, (4) we performed a pull-down assay using an immunoprecipitation method. (5) Then, we calculated the ratio of endogenous SMN to exogenous SMN, which might reflect the oligomerization ability of the mutated SMN1 proteins. Here, we ignored the potential effect of C-terminal tags on the stability and oligomerization ability of mutant proteins.

### 2.3. Plasmid Construction

Preparation of wild-type and mutant-type *SMN1* cDNA sequences was as described previously [22]. Briefly, a wild-type *SMN1* cDNA fragment lacking exon 8 (3′-UTR) was amplified from a normal human fibroblast cDNA library and inserted into the pGEM-T Vector (Promega, Madison, WI, USA) to generate the wild-type *SMN1* cDNA plasmid. To obtain mutant-type *SMN1* cDNA sequences, site-directed mutagenesis was performed using the PrimeSTAR^®^ Mutagenesis Basal Kit (Takara Bio Inc., Kusatsu, Japan).

**Figure 1:**
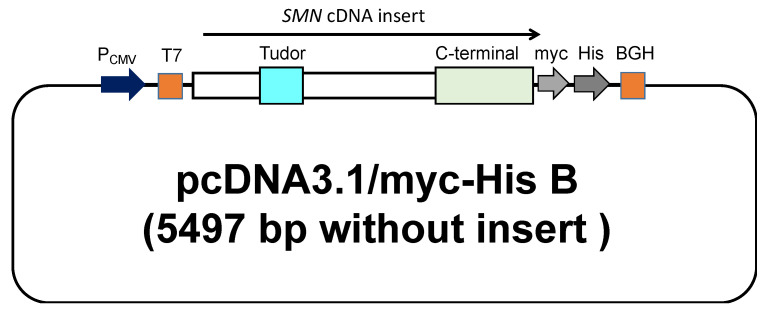
A schematic representation of the plasmid containing the *SMN* cDNA. The wild-type and mutant-type *SMN1* cDNA were inserted into pcDNA™3.1 (Invitrogen, Carlsbad, CA, USA) with a C-terminal myc/His tag. The locations of the Tudor and C-terminal domains are shown in the figure.

Plasmids carrying *SMN* cDNA sequences used in this study were constructed by the following procedure. The wild-type and mutant-type *SMN1* cDNA sequences were subsequently inserted into the expression vector, pcDNA3.1/myc-His B (Invitrogen, Carlsbad, CA, USA). The cloning site of *SMN* cDNA was surrounded by the T7-promoter sequence (T7 in Figure 1) and bovine growth hormone gene polyadenylation signal (BGH in Figure 1). All clones were directly sequenced to confirm the presence of each mutation. The mutant-type *SMN* cDNAs had a mutation in the Tudor or C-terminal domain.

### 2.4. Transfection into HeLa Cells

HeLa cells were maintained in Dulbecco’s Modified Eagle’s medium (DMEM; Wako Pure Chemical Industries, Osaka, Japan) supplemented with 5% fetal bovine serum (Life Technologies, Carlsbad, CA, USA). The cells were transfected with the plasmids containing wild-type and mutant-type *SMN1* cDNA (2.0 μg), using Lipofectamine 2000 (Invitrogen), in accordance with the manufacturer’s instructions. RNA and protein were extracted from the cells harvested at 24 h after transfection. 

A reference plasmid (1 μg) carrying an mCherry sequence encoding a red florescence protein (pmCherry-C1; Clontech Laboratories Inc., Mountain View, CA, USA) was cotransfected with the *SMN* cDNA plasmids to monitor the transfection efficiency. At 24 h after transfection, the transfected cells in a 1 mm^2^ area of each well were captured by the 4× Plan Fluor lens of a BZ-X710 fluorescence microscope (Keyence, Osaka, Japan).

### 2.5. RNA Analysis by RT-PCR

Total RNA was extracted from HeLa cells with TRIzol™ reagent (Thermo Fisher Scientific, Waltham, MA, USA), treated with DNase1 (Invitrogen, Thermo Fisher Scientific) and reverse transcribed by Transcriptor reverse transcriptase (Roche Diagnostics, Manheim, Germany), as previously described [31]. RT-PCR was performed to amplify transcripts from the exogenous *SMN1* gene (the *SMN1* cDNA of the plasmid), endogenous *SMN1* gene (the *SMN1* cDNA of HeLa cells), and the *mCherry* gene of the reference plasmid.

The size of the *SMN* transcript is only ~1.2 kb [8], but the SMN gene is ~20 kb in length [32]. It is easy to differentiate the *SMN* cDNA products from the *SMN* gene products. All constructed plasmids with *SMN1* cDNA carrying a single nucleotide change were almost the same size. In addition, all constructed plasmids were checked by nucleotide sequencing analysis.

The primer sets for exogenous and endogenous *SMN* transcripts were as follows: exogenous *SMN1* transcript: forward primer, 5′-AAT ACG ACT CAC TAT AG-3′ (T7 forward primer in the expression vector) and reverse primer, 5′-TAG AAG GCA CAG TCG AGG-3′ (BGH sequence in the expression vector); and endogenous *SMN* transcript: forward primer, 5′-CTC CCA TAT GTC CAG ATT CTC TTG-3′ (SMNex6F) and reverse primer, 5′-CTA CAA CAC CCT TCT CAC AG-3′ (541C1120; *SMN1* exon 8). 

The *mCherry* transcript was used as a marker of transfection efficiency. The primer set for the *mCherry* transcript was as follows: forward primer, 5′-CGG CAT GGA CGA GCT GTA-3′ and reverse primer, 5′-TCT ACA AAT GTG GTA TGG CTG A-3′. *Glyceraldehyde 3 phosphate dehydrogenase* (*GAPDH*) was used as an internal reference gene. The primer set for *GAPDH* amplification was as follows: forward primer, 5′-GAG TCA ACG GAT TTG GTC GT-3′ and reverse primer, 5′-GAC AAG CTT CCC GTT CTC AG-3′.

### 2.6. SMN1 Protein Quantification by Western Blotting

Protein lysates were prepared using radioimmunoprecipitation assay (RIPA) buffer and brief sonication. A total of 30 μg of homogenized protein samples was subjected to 10% sodium dodecyl sulfate-polyacrylamide gel electrophoresis (SDS-PAGE) and transferred to a polyvinylidene difluoride (PVDF) membrane in iBlot™ gel transfer stack using the iBlot dry blotting system (Invitrogen), as described previously [33]. 

Membranes were blocked with 5% nonfat dry milk and incubated overnight with primary antibodies. Mouse anti-β-actin (1:5000; Sigma-Aldrich, St. Louis, MO, USA) and mouse anti-SMN (1:5000; BD Transduction Laboratories, Franklin Lakes, NJ, USA) primary antibodies were used to detect β-actin and SMN1 proteins, respectively. β-actin was used as an endogenous reference protein and as the relative denominator for Western blotting. Anti-mouse IgG (Cell Signaling #7076 Anti-mouse IgG, HRP-linked) was used as a secondary antibody.

Chemiluminescent signals produced using Immobilon™ Western chemiluminescent HRP substrate (Millipore Corporation, Billerica, MA, USA) were detected using the Bio-Rad ChemiDoc Touch MP imaging system (Bio-Rad Laboratories Inc., Hercules, CA, USA). The signal intensity of the membrane was determined using ImageJ ver. 2.0. Western blotting experiments and signal-intensity analyses were performed in triplicate.

### 2.7. Pull-Down Assay with Immunoprecipitation

HeLa cells transfected with plasmids carrying an *SMN* cDNA sequence were lysed with RIPA lysis buffer (without SDS) (Nacalai Tesque, Kyoto, Japan) for immunoprecipitation. A total of 50 μL of Dynabeads Protein A (Invitrogen) was mixed with mouse monoclonal anti-myc-tag antibody (M-192-3; MBL, Kyoto, Japan) and processed following the manufacturer’s recommendations. An aliquot of 1 mg of total cell lysate was mixed with Dynabead-bound anti-myc-tagged antibody complex. This complex was washed three times with washing buffer and mixed with 20 μL of SDS sample buffer (Nacalai Tesque, Kyoto, Japan). The complex was boiled at 100 °C for 5 min and an aliquot was loaded onto a 10% SDS-PAGE gel. Then, gel electrophoresis and Western blotting analysis were performed to estimate the ability to bind to endogenous FL-SMN1 and/or FL-SMN2 protein (hereinafter referred to as endogenous SMN protein).

### 2.8. Statistics

To measure the abundance of cDNAs or proteins, we determined the intensities of the bands on the gels or membranes. All assays were carried out in triplicate and statistical analyses were performed using Microsoft Excel with add-in software Statcel 3 (The Publisher OMS Ltd., Tokyo, Japan). Results reported as mean ± SD were analyzed by Student’s *t*-test for comparisons between two groups. * *p* < 0.05 and ** *p* < 0.01 were considered to be statistically significant.

## 3. Results

### 3.1. Plasmid Construction and Transfection

#### 3.1.1. Plasmid Construct Carrying Exogenous *SMN* cDNA

**Figure 2:**
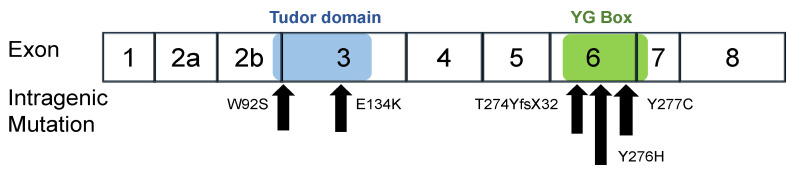
Intragenic mutations in *SMN1.* W92S and E134K are located in the Tudor domain in exon 3. Y277C, Y276H and T274YfsX32 are located in the YG box in exon 6. The positions of the Tudor domain (blue) and YG Box (green) are shown in the figure.

In this study, we constructed six expression plasmid vectors as shown schematically in Figure 1. The plasmids carried the wild-type *SMN1* cDNA and mutant-type *SMN1* cDNA producing mutated SMN1 proteins (W92S, p.Trp92Ser; E134K, p.Glu134Lys; Y276H, p.Tyr276His; Y277C, p.Tyr277Cys; and T274YfsX32, p.Thr274TyrfsX32). Two of the mutations, W92S and E134K, reside in the Tudor domain in exon 3, whereas the other three, Y276H, Y277C, and T274YfsX32, reside in the C-terminal in exon 6 (Figure 2). All mutations are missense mutations except for T274YfsX32, which is a frame-shift mutation. These mutations were previously described elsewhere [19,20,22,27,28,29,30].

#### 3.1.2. Transfection of the Plasmid Containing *SMN1* cDNA

To confirm the successful adjustment of the transfection efficiency of the plasmids containing *SMN* cDNA, HeLa cells were simultaneously transfected with a reference plasmid carrying the red fluorescence expression protein mCherry. The mCherry signals in each well with *SMN1* cDNA-introduced HeLa cells were observed under a fluorescent microscope at 24 h after transfection. The intensity of the mCherry signals was similar among all the wells (Supplementary Figure S1).

### 3.2. SMN Transcript Analysis

#### 3.2.1. Expression of Exogenous *SMN* Transcript in HeLa Cells

**Figure 3:**
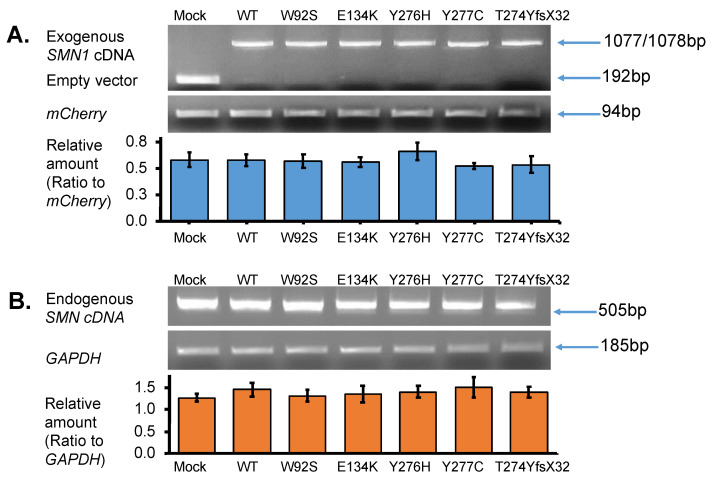
*SMN* transcript analysis. (**A**) Exogenous-*SMN1* cDNA analysis of empty vector (Mock), wild-type (WT) *SMN*, and mutant-type *SMN* cDNA constructs (W92S, E134K, Y276H, Y277C, T274YfsX32) transfected into HeLa cells. One clear band of ~1000 bp was detected for all SMN cDNA constructs. In the Mock, a band of ~200 bp was detected, which was derived from the empty vector transcript. The pmCherry transcript was used for normalization. The amounts of the exogenous SMN1 transcript relative to pmCherry transcript were calculated for comparison. It should be noted that transfection and transcription efficiencies were similar in all the constructs transfected into HeLa cells. (**B**) Endogenous-SMN cDNA analysis of HeLa cells transfected with empty vector (Mock), wild-type (WT) SMN, and mutant-type SMN cDNA constructs (W92S, E134K, Y276H, Y277C, T274YfsX32). Endogenous SMN cDNA encompassing exon 6 to exon 8 was amplified in this study. The amounts of the endogenous SMN relative to GAPDH were calculated for comparison. It should be noted that the introduction of these constructs did not affect the expression of endogenous SMN genes in HeLa cells. Each experiment was performed in triplicate and the data are presented as the mean ± SD.

Total RNA was isolated from the transfected cells to determine exogenous *SMN1* transcript levels, as described in the methods section. PCR amplification was performed using vector-specific primers (or primers for T7 promotor sequence and BGH gene polyadenylation signal sequence) to confirm the presence of the correct insert. Analysis of the RT-PCR products on a 2% agarose gel disclosed a lower band (192 bp) detected in the empty vector-transfected cells (Mock) (Figure 3A upper panel). In contrast, one clear band (either 1077 or 1078 bp) was observed in the samples with WT, W92S, E134K, Y276H, Y277C, and T274YfsX32 cDNA. This band was determined as the exogenous *SMN1* transcript (Figure 3A upper panel). These results clearly indicated that insertion of the cDNA fragment into the expression vector as well as transfection of the plasmids into the HeLa cells was successful. 

#### 3.2.2. Expression of Endogenous *SMN* Transcript in HeLa Cells

We next analyzed the endogenous *SMN* transcript by amplifying the region between exon 6 and exon 8 of the *SMN* transcript using a primer set described in the method section. Since exon 8 is not included in the exogenous *SMN* sequence, we could securely amplify only the endogenous *SMN* transcript. It should be noted that the endogenous *SMN* transcript was produced by *SMN1* and *SMN2* in HeLa cells. From these results, the band intensities of endogenous *SMN* transcript were similar in each well with *SMN1* cDNA-introduced HeLa cells (Figure 3B upper panel). 

#### 3.2.3. Quantification of Exogenous and Endogenous *SMN* Transcripts by RT-PCR

To compare the levels of exogenous and endogenous *SMN1* transcripts among samples, we performed semi-quantitative RT-PCR. *mCherry* and *GAPDH* transcripts were amplified in all samples and used as reference genes for normalization. The abundance of the exogenous *SMN1* transcript relative to that of the *mCherry* transcript was measured and displayed in a bar graph (Figure 3A lower panel). From the results, there were no significant differences in transcript levels among the samples, indicating that equivalent amounts of plasmid were delivered to the nuclei of HeLa cells and were properly transcribed to produce the exogenous *SMN1* transcript. 

Similarly, the abundance of the endogenous *SMN* transcript relative to that of *GAPDH* was also evaluated and presented in a bar graph (Figure 3B lower panel). There were no significant differences in transcript levels between the samples. This indicated that the overexpression of the plasmids produced no feedback effect in the endogenous *SMN* in HeLa cells at the transcription level. 

### 3.3. SMN1 Protein Analysis 

#### 3.3.1. Western Blotting Analysis with Anti-SMN Antibody

**Figure 4:**
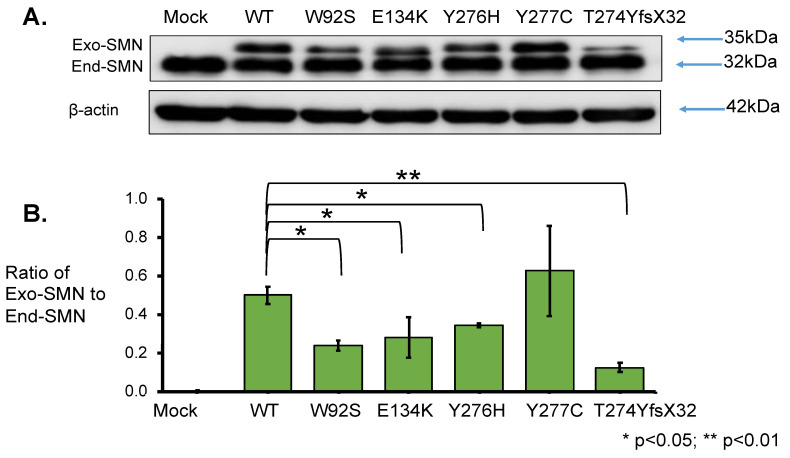
SMN1 protein analysis. (**A**) Western blotting using anti-SMN antibody. HeLa cells were transfected with empty vector (Mock), wild-type (WT) *SMN* and mutant-type *SMN* cDNA constructs (W92S, E134K, Y276H, Y277C, T274YfsX32). Western blotting of the cell lysates was performed with anti-SMN antibody. The bands corresponding to the exogenous and endogenous SMN are shown in the figure, as Exo-SMN and End-SMN, respectively. β-Actin was used as an internal control. (**B**) Relative amounts of exogenous SMN. The ratios of exogenous SMN relative to endogenous SMN were calculated for comparison after normalization with β-actin. Each experiment was performed in triplicate and the data are presented as the mean ± SD. * *p* < 0.05 and ** *p* < 0.01.

To elucidate the effect of mutation on protein stability, SMN1 protein levels were determined. Total lysates for Western blotting were prepared from the cells harvested at 24 h after transfection. From the results obtained using anti-SMN antibody, two clear bands of approximately 35 kDa and 32 kDa were observed; the upper band represented exogenous SMN, while the lower band represented endogenous SMN (Figure 4A). The band intensities of endogenous SMN proteins were similar in all samples. Western blotting with anti-β-actin antibody also showed similar band intensities in all samples (Figure 4A).

However, the intensity of exogenous SMN1 proteins varied among the samples. We evaluated the relative amounts of exogenous SMN1 and endogenous SMN proteins after normalization to β-actin (Supplementary Table S1). The mutant SMN1 protein levels with W92S, E134K, Y276H, and T274YfsX32 were significantly reduced compared with that of the wild-type (WT) SMN1 protein. However, the mutant SMN1 protein level with Y277C was not significantly different from that of the wild-type (WT) SMN1 protein (Supplementary Table S1). 

Here, we calculated the ratio of exogenous SMN1 protein level to endogenous SMN protein level (ratio of Exo-SMN to End-SMN in Figure 4B, Supplementary Table S1). The findings in descending order of protein level (mean value) of exogenous SMN1 proteins were as follows: Y277C, WT, Y276H, E134K, W92S and T274YfsX32. The mutated SMN1 protein with T274YfsX32 showed the lowest level among the exogenous SMN1 proteins. If the translation efficiency was the same among the exogenous SMN1 transcripts, this suggested that SMN1 protein with T274YfsX32 was the most unstable protein. 

#### 3.3.2. Pull-Down Assay with Immunoprecipitation 

**Figure 5:**
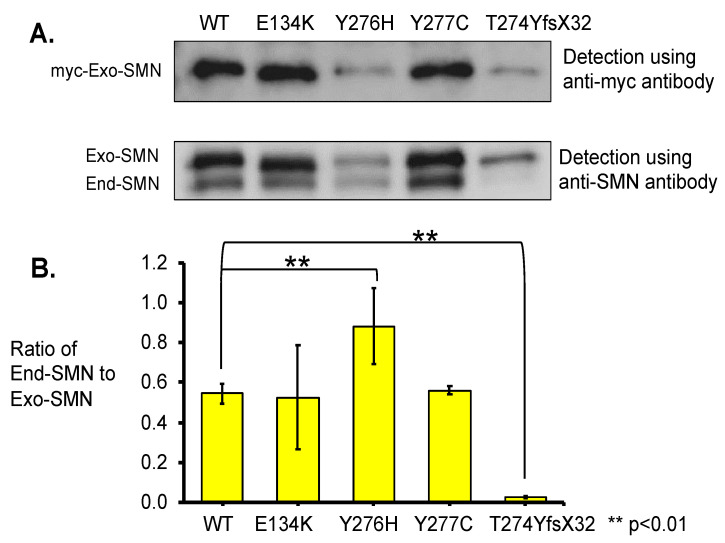
Immunoprecipitation analysis. (**A**) Total lysates of the HeLa cells transfected with wild-type (WT) *SMN* and mutant-type *SMN* cDNA constructs (WT, E134K, Y276H, Y277C, T274YfsX32) were immunoprecipitated with anti-myc-tag and analyzed with anti-myc and anti-SMN antibodies. When anti-SMN antibodies were used, two bands corresponding to the exogenous and endogenous SMN were indicated. The exogenous and endogenous SMN are indicated as Exo-SMN and End-SMN, respectively. (**B**) Relative amounts of endogenous SMN. The ratios of endogenous SMN relative to exogenous SMN were calculated for comparison. Each experiment was performed in triplicate and the data are presented as the mean ± SD. ** *p* < 0.01.

To estimate the oligomerization ability of the mutated SMN1 proteins with a mutation in the C-terminal domain, we performed immunoprecipitation analyses of exogenous SMN1 proteins (or myc-tagged SMN1 proteins) using anti-myc-tag antibody (Figure 5). The immunoprecipitated products were analyzed with both the anti-myc-tag and the anti-SMN antibodies. In this assay, one mutated SMN1 protein with a mutation in the Tudor domain, E134K, was included as a reference because it has already been reported that this mutation does not affect self-association or oligomerization [11]. In this assay, myc-tagged SMN protein with W92S was not included because enough amount of immunoprecipitated products were not obtained because of unknown reasons.

Blotting with the anti-myc-tag antibody indicated differences in band intensity among the exogenous SMN1 proteins. The samples of wild-type (WT) SMN1 protein and mutated SMN1 proteins with E134K and Y277C showed clear bands with high intensity, while the samples of mutated SMN1 proteins with Y276H and T274YfsX32 showed faint bands with the lowest intensity (Figure 5A). 

Blotting with the anti-SMN antibody indicated two clear bands for the exogenous and endogenous SMN (Figure 5A). The samples of wild-type (WT) SMN1 protein and mutated SMN1 proteins with E134K and Y277C showed clear bands of the exogenous SMN1 and endogenous SMN proteins. However, the sample of mutated SMN1 protein with Y276H showed faint bands of the exogenous SMN1 and endogenous SMN proteins, although these two bands were faint, they showed that both proteins definitely existed. The last sample of mutated SMN1 protein with T274YfsX32 showed a faint band of the exogenous SMN1 protein but almost no band of the endogenous SMN protein. 

Here, we calculated the ratio of endogenous SMN protein level to exogenous SMN1 protein level (ratio of End-SMN to Exo-SMN in Figure 5B, Supplementary Table S2) in this pull-down assay. The findings arranged in descending order of the ratios (mean value) were as follows: Y276H, Y277C, WT, E134K, and T274YfsX32. The ratio value of T274YfsX32 was extremely low, 0.02, while those of the other mutations exceeded 0.5 (Supplementary Table S2). 

## 4. Discussion

### 4.1. SMA Patients with an Intragenic Mutation in the Retaining SMN1 Allele

The clinical severity of SMA correlates mainly with the copy number of the *SMN2* genes [6]. Infants with the most severe form of disease (type 0) usually have only one copy of *SMN2*, while infants with SMA type 1 usually have two or three copies. SMA type 2 is usually associated with three copies, type 3 patients have three to four copies, and patients with type 4 usually have four copies or more. Based on these findings, it has been considered that the existence of multiple copies of *SMN2* may compensate for the deletion of the *SMN1* gene, modifying the clinical severity of the disease. 

However, in a clinical context, some exceptional cases have been encountered, including patients with a more severe phenotype but a higher copy number of *SMN2* or with a milder phenotype but a lower copy number of *SMN2*. We reported two type 1 patients with three *SMN2* copies who showed a more severe phenotype than expected [20], and two type 3 patients with only a single copy of *SMN2* who could walk independently [19]. These patients were heterozygous for deletion of one *SMN1* allele and had an intragenic mutation in the retained *SMN1* allele.

*SMN2* copy number is not always associated with the clinical severity of SMA patients, especially those with an intragenic mutation in the retained *SMN1* allele [19]. The locations and types of intragenic *SMN1* mutations may be more critical in determining the clinical phenotype than *SMN2* copy number [15]. In this study, to clarify why the locations and types of intragenic *SMN1* mutations are determinants of SMA phenotype, we attempted to determine the stability and oligomerization ability of the mutated SMN1 proteins in some SMA patients.

### 4.2. Stability of the Mutated SMN1 Proteins

We determined the expression levels of *SMN1* transcript and SMN1 protein derived from the plasmid construct carrying mutant-type *SMN1* cDNA in HeLa cells. The transcript levels from wild-type and mutant-type *SMN* cDNA were almost the same (Figure 3). However, the levels of protein generated from mutant-type *SMN* cDNA were generally lower than those from wild-type *SMN* cDNA (Figure 4). As long as the translation from transcripts to proteins proceeds smoothly, our results would indicate that, generally, mutated SMN1 protein is less stable than wild-type (WT) SMN1 protein. It is already known that SMN1 protein is degraded by the ubiquitin–proteasome system [11]. Taking these findings together, our study suggested that the ubiquitination of mutated SMN1 proteins might proceed rapidly, followed by their proteasomal degradation. However, in this study, we assumed that the translation ratio was the same for all the SMN1 mutated proteins. To show more accurate results of protein stability, it might be necessary to stop new production of SMN1 protein by cycloheximide treatment.

However, the stability of SMN1 proteins may vary in a mutation-dependent manner (Figure 4, Supplementary Table S1). The stability of the SMN1 protein with Y277C (c.830 A > G, missense mutation) was almost the same as that of the wild-type (WT) SMN1 protein. Meanwhile, the stabilities of SMN1 protein with W92S (c.275 G > C, missense mutation), SMN1 protein with E134K (c.400 G > A, missense mutation), and SMN1 protein with Y276H (c.826 T > C, missense mutation) were about half that of wild-type (WT) SMN1 protein. The stability of mutated SMN1 protein with T274YfsX32 (c.819_820 insT, frame-shift mutation) was much less than that of other SMN1 proteins. Thus, in this study, we confirmed that the locations and types of intragenic *SMN1* mutation may determine the stability of the protein products. 

SMN1 stability is influenced by complex formation and oligomerization [11]. According to the binding assays with overexpressed mutated proteins, W92S or E134K severely reduced the ability of SMN1 to interact with Sm proteins and fibrillarin [20,34,35]. Although Burnett et al. showed that mutated SMN1 protein with E134K did not show a shorter half-life than FL-SMN1 protein, they also showed that SMN complex formation with Gemin3, 5, or 6 led to resistance to degradation [11]. Our data demonstrated that mutated SMN1 protein with E134K (and W92S) was easily degraded, suggesting that the lack of complex formation with Sm proteins, fibrillarin, and other proteins, may be related to instability to some degree [22]. Correlations between oligomerization ability and stability of SMN1 protein will be discussed in the next subsection. Although the endogenous level of SMN protein in patient cells may vary among patients, we can say the presence of full-length SMN2 protein may be essential for the survival of the patients with SMN1 defects. Thus, we expect the possible function of a heterodimer of mutated SMN1 protein and FL-SMN2 protein in patients’ cells.

### 4.3. Oligomerization of SMN Proteins 

SMN protein oligomerizes with itself, and the modular oligomerization motif is within *SMN1* exon 6 [23]. The oligomeric states of SMN protein range from dimers to octamers through the so-called YG-box domain (residues 254–280), which contains a (YxxG)_3_ motif [34]. SMN proteins form helical oligomers with tetrameric, pentameric, and heptameric components (referred to as “glycine zippers”) [34]. According to the molecular genetic analysis of SMA patients with an intragenic mutation, nearly half of the known missense mutations are located in the YG-box domain [27]. Oligomerization failure may result in rapid degradation of the SMN protein and hence low protein levels [11]. However, all mutations located in the YG-box may not disrupt oligomerization to the same degree.

To estimate the oligomerization ability of mutated SMN1 proteins, we performed an immunoprecipitation assay to determine the ability of exogenous, mutated SMN1 proteins to bind to endogenous SMN proteins in HeLa cells. This analysis would not detect whether the mutated SMN1 protein can form a homodimer complex or determine what percentage of the mutated SMN1 protein can participate in formation of a heterodimer complex. Even so, detecting the ability of mutated SMN1 proteins to bind to endogenous SMN means that the mutated SMN1 proteins have some oligomerization ability, suggesting that the mutated SMN1 proteins can form a heterodimer with FL-SMN2 protein in patients’ cells.

Based on the results of our immunoprecipitation study, mutated *SMN1* proteins with Y276H or Y277H did not show a lower ability to bind to endogenous SMN protein compared with the wild-type (WT) or E134K proteins. In contrast, mutated *SMN1* protein with T274YfsX32 showed almost no ability to bind to endogenous SMN protein in HeLa cells. These results suggested that the oligomerization ability of mutated SMN1 proteins with Y276H or Y277H was maintained to some degree, but that of mutated SMN1 proteins with T274YfsX32 was almost completely lost.

Since the insertion of an additional T in exon 6 may lead to a shift of the open reading frame, the amino acid sequence of the C-terminal of the mutated SMN1 protein with T274YfsX32 differed from those of other SMN1 proteins [22], indicating that the mutation destroyed the YG-box and eliminated the sequence of exon 7. Thus, we had expected the complete loss of oligomerization ability in the mutated SMN1 protein with T274YfsX32. In this study, only the frameshift mutation was shown to disrupt oligomerization. However, Lorson et al. described high oligomerization defect in severely affected patients with Y272C and G279V in the *SMN1* gene [1]. According to Gupta et al. (2021) [35], tyrosine (Y) at position 272 and glycine (G) at position 279 are strictly conserved and highly significant residues across species. Taken together with our results, a single mutation in the YG-box may destroy oligomerization ability to various degrees, but mutations which replace the conserved residues may disrupt oligomerization severely. 

### 4.4. Clinical Phenotype of the Patients with an Intragenic Mutation

Two hypotheses have been proposed to explain the pathogenesis of SMA [27]. The first is that small nuclear ribonucleoprotein (snRNP) assembly is disrupted by SMN protein defects, preventing the splicing of a group of genes that play important roles in motor neurons. The second is that, in SMA motor neurons, the special function of SMN proteins is destroyed by their mutations. 

The first hypothesis is supported by many studies on snRNP biogenesis [36]. Reduced snRNP levels due to dysfunction or a low level of SMN protein would be expected to cause splicing defects and changes in mRNA expression [37]. The second is supported by intensive studies of the RNA-binding protein HuD [38,39,40,41]. SMN–HuD complexes are essential for normal motoneuron development [41]. Disrupting the interaction between these two proteins can cause SMA phenotypes [41]. However, regardless of which of these hypotheses is true, oligomerization is a key element for SMN function [40,42].

Recently, Iyer et al. reported that heteromeric SMN complex of mutated SMN and FL-SMN proteins is functional and sufficient to rescue snRNP assembly and motor neuron function, and to rescue SMA-model mice [43]. According to them, mild SMN missense alleles were completely nonfunctional in the absence of wild-type SMN. Their findings led us to the idea that the oligomerization ability of the mutated SMN1 proteins may determine the clinical severity of the patients with an intragenic mutation.

A patient with Y276H (Patient 04) presented with typical clinical features of SMA type 2 [24]. She showed no particular findings in the neonatal period. At 8 months old, she had not yet achieved the expected head-control milestone. At 3 years old, she could sit with support, but her head control was incomplete. She carried two copies of *SMN2* (Table 1). The stability of the mutated SMN1 protein (ratio of Exo-SMN/End-SMN in Western blotting analysis) was 69% of the wild-type (WT) SMN protein stability (Supplementary Table S1), and the ability to bind to endogenous SMN protein (ratio of End-SMN/Exo-SMN in the immunoprecipitation experiment followed by Western blotting) was 153% of the wild-type (WT) SMN protein ability (Supplementary Table S2).

Surprisingly, an SMA type 2/3 patient with Y277C (Patient 05) presented with a much milder phenotype [19]. Based on the motor milestones she achieved, she was a patient with “type 2 close to type 3” SMA (Table 1). She could sit unaided and stand while using a wall or table for support. However, she rapidly lost such abilities at 2 years old. She carried only one copy of *SMN2* (Table 1). The stability of the mutated SMN1 protein (ratio of Exo-SMN/End-SMN in Western blotting analysis) was 125% of the wild-type (WT) SMN protein stability (Supplementary Table S1), and the ability to bind to endogenous SMN protein (ratio of End-SMN/Exo-SMN in the immunoprecipitation experiment followed by Western blotting) was 110%, almost the same as the wild-type (WT) SMN protein stability (Supplementary Table S2).

In contrast to the above-mentioned patients, an SMA type 1 patient with T274YfsX32 in our study (Patient 06) presented with the most severe phenotype [19,29]. The patient was a male who showed dyspnea with paradoxical breathing and generalized hypotonia shortly after birth. His respiratory condition gradually deteriorated, and he underwent a permanent tracheostomy at 6 months. He carried two copies of *SMN2* (Table 1). The stability of the mutated SMN1 protein (ratio of Exo-SMN/End-SMN in Western blotting analysis) was 25% of the wild-type (WT) SMN protein stability (Supplementary Table S1), and the ability to bind to endogenous SMN protein (ratio of End-SMN/Exo-SMN in the immunoprecipitation experiment followed by Western blotting) was 4% of the wild-type (WT) SMN protein ability (Supplementary Table S2). The extremely low value of the ratio of End-SMN/Exo-SMN in this patient may suggest a very low possibility of heterodimer complex formation with FL-SMN2 protein.

**Figure 6:**
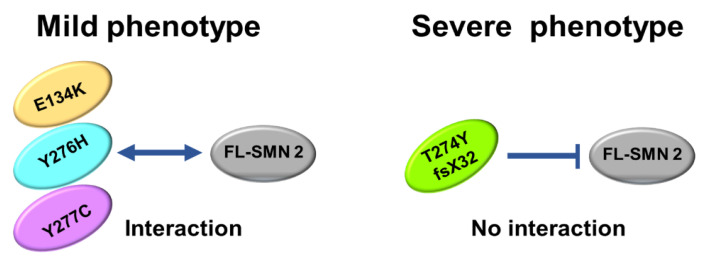
Scheme of oligomerization. E134K, Y276H, and Y277C mutations could maintain oligomerization ability to some degree, leading to interaction with FL-SMN2 (or full-length SMN2 protein). However, T274Yfs mutation almost completely lost oligomerization ability, which blocked interaction with FL-SMN2.

The number of patients analyzed in this study was very limited, but our analysis suggested that the oligomerization ability of the mutated SMN1 protein, which is linked to interaction with FL-SMN2 protein, may be a determinant of the clinical severity of SMA (Figure 6). 

## 5. Conclusions

In conclusion of this study, the stability and oligomerization ability of mutated SMN1 protein may determine the protein stability and may be associated with the clinical severity of SMA caused by an intragenic *SMN1* mutation.

According to our series of studies with SMA patients carrying an intragenic *SMN1* mutation, there are at least three determinants of clinical phenotype of the SMA patients with an intragenic SMN mutation; (1) complex formation with partner proteins bound to mutated SMN1 proteins [20], (2) stability of mutated SMN1 proteins in [22] and this study and (3) oligomerization ability of mutated SMN1 proteins (this study). Complex formation, stability and oligomerization of the SMN protein are closely intertwined and cannot be considered separately. However, new treatment strategies targeting these three factors should be explored to treat SMA patients with an intragenic *SMN1* mutation.

## Figures and Tables

**Table 1:** Clinical information of the patients with intragenic mutation.

Patient	Mutation Name	Clinical Subtype	*SMN1* Copy Number	*SMN2* Copy Number	Mutation Type	Nucleotide Change	Amino Acid Change	Exon Domain	Ref.
01 02	W92S	1	1	3	Missense	c.275 G > C	p.Trp92Ser	Ex3 Tudor	[20,22]
03	E134K	2	1	2	Missense	c.400 G > A	p.Glu134Lys	Ex3 Tudor	[27,30]
04	Y276H	2	1	2	Missense	c.826 T > C	p.Tyr276His	Ex6 C-term. *	[28]
05	Y277C	2/3	1	1	Missense	c.830 A > G	p.Tyr277Cys	Ex6 C-term. *	[19]
06	T274Y fsX32	1	1	2	Frame shift	c.819_820 insT	p.Thr274TyrfsX32	Ex6 C-term. *	[19,22,29]

* C-term; C-terminal, Notes about clinical phenotype of patients 1 and 2 (supplementary notes).

## Data Availability

The data presented in this paper are available on request from the corresponding author.

## Supplementary Materials

The following supporting information can be downloaded at: https://www.mdpi.com/article/10.3390/genes13020205/s1, Figure S1: Adjustment of transfection efficiency of the plasmid containing SMN1 cDNA.; Table S1: SMN protein amounts quantitated by Western blotting analysis and ratios of Exo-SMN/End-SMN; Table S2: Immunoprecipitated protein amounts quantitated by Western blotting analysis and ratios of End-SMN /Exo-SMN; Supplementary notes.
