# Supplementary material for: Stability and Oligomerization of Mutated SMN Protein Determine Clinical Severity of Spinal Muscular Atrophy
Source: Genes (Basel). 2022 Jan 24;13(2):205. doi: 10.3390/genes13020205 (PMC8872419; doi:10.3390/genes13020205)
Supplement: Supplementary file 1 [file genes-13-00205-s001.zip › genes-1541818-supplementary.pdf]

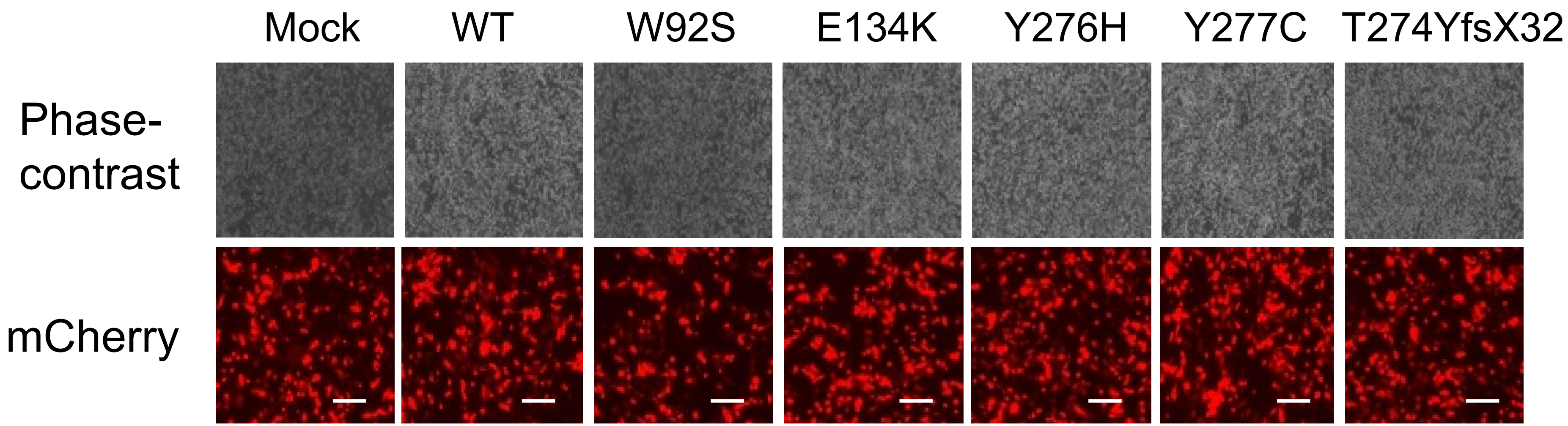

Figure S1: Adjustment of transfection efficiency of the plasmid containing *SMN1* cDNA. Red florescence signal (mCherry) in HeLa cells transfected with empty vector (Mock), wild-type (WT) and mutant-type SMN cDNA (W92S, E134K, Y276H, Y277C, T274YfsX32) were observed under BZ - X710 fluorescence microscope (Keyence, Osaka, Japan). The white bar indicates 100μM.

Table S1: SMN protein amounts quantitated by Western blotting analysis and ratios of Exo-SMN/End-SMN

	WT	W92S	E134K	Y276H	Y277C	T274Yfs
Exo-SMN	1.084	0.469	0.545	0.646	1.066	0.241
End-SMN	2.163	1.963	1.931	1.864	1.698	1.902
Exo-SMN /End-SMN	0.501 (100%)	0.239 (48%)	0.282 (56%)	0.347 (69%)	0.628 (125%)	0.127 (25%)

End-SMN value represents mean amount of endogenous SMN protein.
Exo-SMN value represents mean amount of exogenous SMN1 protein.
A ratio of Exo-SMN/End-SMN represents estimated SMN protein stability.

Table S2: Immunoprecipitated protein amounts quantitated by Western blotting analysis and ratios of End-SMN /Exo-SMN

	WT	E134K	Y276H	Y277C	T274Yfs
End-SMN	5.690	4.953	3.136	6.377	0.070
Exo-SMN	10.459	9.436	3.551	11.397	2.893
End-SMN /Exo-SMN	0.544 (100%)	0.525 (96%)	0.883 (153%)	0.559 (110%)	0.024 (4%)

End-SMN value represents mean amount of endogenous SMN protein.
Exo-SMN value represents mean amount of exogenous SMN1 protein.
A ratio of End-SMN/Exo-SMN represents estimated oligomerization ability.

Supplementary information

Notes about clinical phenotype of patients 1 and 2: The W92S and E134K mutation both reside in the exon 3-tudor domain which has been reported to be important in Sm-ring assembly [23]. The patients with W92S mutation in this study, who had 3 *SMN2* copy numbers, showed a more severe phenotype than the patient with E134K mutation who had 2 *SMN2* copies. There may be unknown factors associated with the W92S mutation, which are not affected by the E134K mutation.
